# Development and application of a self-transcendence enhancement program for the well-being of elderly women living alone in Korea

**DOI:** 10.4069/kjwhn.2021.06.07

**Published:** 2021-06-24

**Authors:** Sun-Mi Kim, Sukhee Ahn

**Affiliations:** 1 Medical insurance review, Chungnam National University Hospital, Daejeon, Korea; 2 College of Nursing, Chungnam National University, Daejeon, Korea

**Keywords:** Aged, Depression, Female, Personal satisfaction, Psychological adaptation

## Abstract

**Purpose:**

The purpose of this study was to develop a self-transcendence enhancement program and examine its effect on self-transcendence, spiritual well-being, and psychological well-being in elderly women who live alone.

**Methods:**

A self-transcendence enhancement program was developed through theory, literature review, and in-depth interviews. The theoretical framework came from the Psychoeducational Approach to Transcendence and Health intervention model based on Reed’s middle-range theory of self-transcendence. The program consisted of multiple modalities in a structured, theory-based program lasting for eight weekly sessions. Using a single-group pretest-posttest design, the program was tested on a group of 40 elderly women aged 75 to 84 years living alone in Daejeon, South Korea. Participants completed self-reported study questionnaires before and after the program at the elderly welfare center. Data were analyzed using SPSS version 24.0, with significance level set at .05. Paired t-test was used to compare mean differences before and after the program.

**Results:**

The mean age of the study participants was 79.1 years. After completing the program, the participants showed higher levels of self-transcendence (t=8.78, *p*<.001), overall spiritual well-being (t=8.30, *p*=.002), religious spiritual well-being (t=1.79, *p*=.040), existential spiritual well-being (t=6.75, *p*=.002), and positive affect (t=3.77, *p*=.001) than they did before the program. They also reported lower levels of depression (t=–7.59, *p*<.001) and negative affect (t=–6.15, *p*<.001).

**Conclusion:**

The self-transcendence enhancement program developed in this study may be effective for improving the level of self-transcendence in elderly women living alone and helping them to attain spiritual and psychological well-being.

## Introduction

### Background/rationale

In terms of well-being, elderly women who live alone appear to be a more vulnerable group than other demographic groups in South Korea (hereafter, Korea) [1,2]. According to studies of the well-being of elderly women who lived alone, 61.7% belonged to the depressed group [1], with subjective well-being also showing poor results for life satisfaction and positive emotions in addition to above-average negative emotions [3]. In addition, while the spiritual well-being of elderly women living alone was found to be slightly above average, it was lower than that of elderly people who lived alone overall [2,4]. The psychological and spiritual well-being of elderly women living alone is relatively poor due to interactions between various circumstances making them vulnerable. As such, elderly women living alone are often in a state of crisis [5,6], with vulnerable living conditions and limited support. The healing of resentment and depression felt by elderly women living alone is deeply related to the psychological and spiritual concepts of gratitude and forgiveness [7,8]. Thus, the need to pursue well-being beyond one’s current vulnerable situation is higher for elderly women living alone than for other age groups. It may be possible to reach a state of well-being through self-transcendence by changing one’s own inner life and perspective. Therefore, interventions for psychological and spiritual well-being are needed for elderly women in a position of vulnerability [9].

Self-transcendence is the core concept of self-transcendence theory, which was developed by the nursing theorist Reed [9]. It refers to the process of expanding one’s boundaries to deeper and higher dimensions to overcome a personal crisis using the following four components; a deep understanding of oneself, expansion of one’s interpersonal relationships through interactions with others, connection with a dimension beyond the typically perceived world, and integration of the past and the future into the present [9]. Programs using the concept of self-transcendence improved psychological well-being by reducing depression among elderly people in a nursing home in one study [10] and, in another study, improved the subjective well-being of community-dwelling elderly women [11]. Self-transcendence programs have also helped to promote the psychological and spiritual well-being of elderly women and breast cancer patients living alone in times of crisis [12,13]. Interventions to improve the well-being of elderly women living alone have usually focused on the physical, psychological, and social problems caused by the vulnerability of this population. Some interventions to deal with physical and psychological health issues among this population have included urinary incontinence prevention exercises, art therapy, and group play therapy [14-16]; subjective well-being and depression reduction [3]; depression and physical health improvement [17]; and self-esteem and interpersonal relations [18]. However, despite the many difficult life events and health experiences had by elderly women living alone, it is difficult to find programs that promote their psychological and spiritual well-being, including self-transcendence.

### Objectives

**Figure 1.**
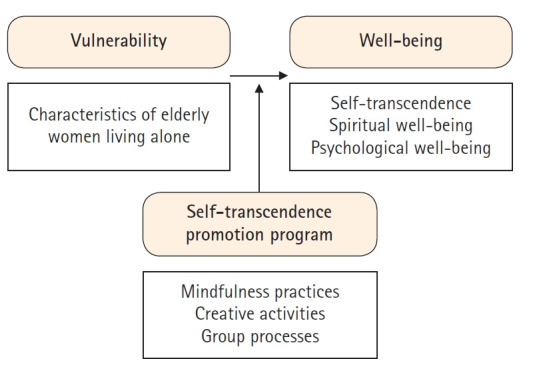
Conceptual framework of the study.

This study was based on Reed’s self-transcendence theory [9], which directly addresses the life experiences and the demand for self-transcendence of elderly women living alone. A conceptual framework for this study was created to help develop a self-transcendence enhancement program and evaluate its applicability (Figure 1). The program in this study can be implemented in practice to improve the psychological and spiritual well-being of elderly women living alone. There were two specific objectives when designing the program: first, to develop a self-transcendence enhancement program based on self-transcendence theory by identifying attributes related to well-being experienced by elderly women living alone; and second, to examine the effects of the program on self-transcendence, spiritual well-being, and psychological well-being (depression, life satisfaction, positive affect, negative affect) of elderly women living alone. Three hypotheses were set: *first*, elderly women living alone who participated in the self-transcendence enhancement program would have a stronger sense of self-transcendence than they did before participating in the program; *second*, elderly women living alone who participated in the self-transcendence enhancement program would have greater spiritual well-being than before; and *third*, elderly women living alone who participated in the self-transcendence enhancement program would have greater psychological well-being than before. Specifically, decreased depression and increased life satisfaction, positive affect, and negative affect were anticipated.

## Methods

Ethics statement: This study was approved by the Institutional Review Board of Chungnam National University (201907-SB-106-01). Informed consent was obtained from the participants.

### Study design

This study is a single-group pretest-posttest experimental study.

### Participants

The participants of this study were elderly Korean women living alone. The researcher (first author) selected two senior citizens centers using convenience sampling based on a report on the current status of elderly women living alone, issued by the Ministry of Health and Welfare [19]. Participants were chosen from those who voluntarily agreed to participate in the study. The inclusion criteria were Korean women living alone, who were between 75 to 84 years of age, and understood the purpose of the study. They also had to be able to read, write, listen, comprehend, and express themselves. The participants were further limited to those who had not experienced a similar self-transcendence program consisting of training, meditation, self-reflection, creative activities, and group discussions, and who could attend more than 75% of the eight sessions. Women aged 75 to 84 years old specifically were chosen as participants since this was the age range with the highest proportion of women living alone [2,20]. Furthermore, elderly women above the age of 85 years tend to consider changes in well-being as a natural phenomenon due to advanced age and accept their present lives more positively [21]. The exclusion criteria were those with expected difficulty participating in the program and survey, based on self-report of any of the following: due to severe chronic diseases (grade 2 or higher), mental illness, cognitive impairment, visual impairment, or hearing impairment.

### Study size

**Figure 2.**
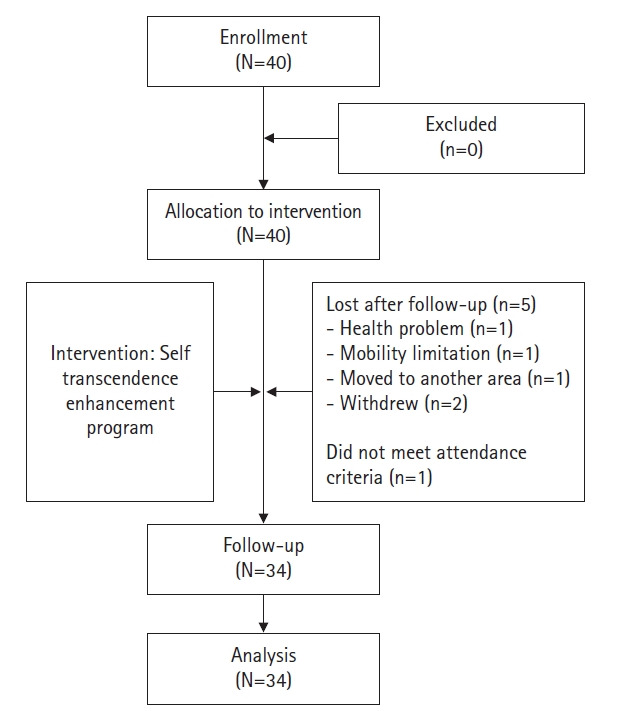
Flow chart of the study.

The effect size of the self-transcendence variable (d=.70) and the effect size of the depression variable (d=.45) suggested by Kim and Kim’s [22] self-transcendence program were considered, and the lower of the two values was selected for this study. Thus, the effect size was set to d=.45, and with a significance level of α=.05 and power of .80, the minimum sample size required for a one-sided paired t-test was found to be 32 participants. A total of 40 people were recruited for the final sample size, in estimation of a potential 20% dropout rate in the course of the 8-week program. By the end of the program, there were 34 participants (85% retention rate) (Figure 2).

### Development of the self-transcendence enhancement program

#### Analysis of the literature and content of in-depth interviews

Interventional studies that applied Reed’s concept of self-transcendence [9] were selected by searching literature databases including KoreaMed, Korea Citation Index, PubMed, and CINAHL. A study that reported improved self-transcendence of community-dwelling elderly women [11] provided a basis for organizing the eight topics included in this study’s program across eight 1-hour sessions over a total of eight weeks. That study’s framework served as a starting point for the introductions, development, conclusions, and family activities included in our program. In addition, single sessions in our program were designed to include 10 minutes for introductions, 40 minutes for development, and 10 minutes for conclusions, based on the time breakdown of a self-transcendence enhancement program for drug addicts [22]. Based on another study that conducted a social support activity program for the elderly [10], the class size for the program was small (limited to 6-7 people) to encourage active communication, support, and reassurance.

In-depth interviews and qualitative content analysis were conducted to select a program theme that reflected the specific aspects related to self-transcendence among elderly women. In-depth interviews were conducted with nine women who voluntarily participated, using the same inclusion criteria. These women were independent of the larger study and interviewed twice (over 2 weeks) from November 2 to 16, 2019. The questionnaire items included in the in-depth interviews focused on the four dimensions of self-transcendence (individual, interpersonal, individual, and temporal) for elderly women living alone (Supplementary data). One-on-one interviews were conducted and lasted about 1 hour per session.

The qualitative analysis involved applying the three stages of inductive content analysis devised by Elo and Kyngäs [23]—open coding, categorization, and abstraction—to understand the perspectives on life of elderly women living alone and the implicit categories related to their self-transcendence. The categorizations included ambivalence toward oneself, the limits of interpersonal relationships, negative self-awareness toward aging, negative emotions toward life, the desire to pursue spirituality, awareness of a better life, and the disharmony between one’s desires for the rest of life and the reality. These formed eight meaning clusters at the final level of abstraction: self-expression and respect, overcoming feelings of regret, caring, forgiving, building relationships, living an altruistic life, having a good death, and finding purpose and meaning in life.

#### Program composition

**Figure 3. f3-kjwhn-2021-06-07:**
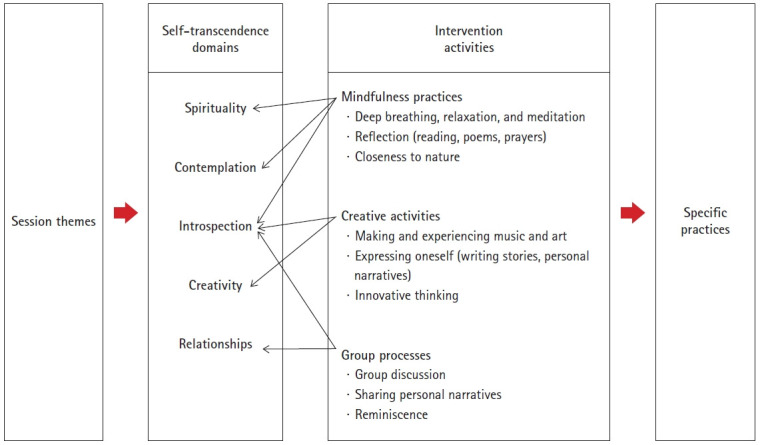
Framework of the self-transcendence enhancement program in the study.

Based on the eight meaning clusters, intervention activities were devised and concrete activities were created. The themes of the program were based on the eight meaning clusters related to the self-transcendence needs of elderly women living alone, with a focus on the expansion of temporal boundaries (Table 1). The intervention activities in this study were based on the five self-transcendence domains and three intervention activities suggested in the model of the Psychoeducational Approach to Transcendence and Health intervention method [24], which was developed by modifying the concept of self-transcendence. The five transcendental areas were spirituality, contemplation, introspection, creativity, and relationships. The three intervention activities were mindfulness practice, creative activities, and group processes (Figure 3). The specific activities included in the final program were introduction, development, conclusion, and family activities. Body scan meditation (a mindfulness activity that includes deep breathing, relaxation, and meditation) was conducted during each session to foster mindfulness and improve spirituality, contemplation, and self-reflection. The family activities that were practiced during the week following each session consisted of activities to practice mindfulness [11] to improve spirituality, contemplation, and self-reflection.

#### Validity of the program

To ensure the validity of the program, this study was revised once and supplemented after receiving advice from a nursing professor and geriatric and psychiatric advanced practice nurses. A preliminary survey was conducted during an 11-day period with six elderly women who lived alone in a senior citizen center in Daejeon, Korea, regarding the program’s procedures to verify the completeness of its content and composition and to identify any unexpected problems. As a result of this preliminary survey, only body scan meditation in the sitting position was included in the final program due to the survey respondents’ opinion that the basic yoga movement included as a mindfulness activity could cause strain on the joints of program participants (Table 1).

#### Operation of the self-transcendence enhancement program for elderly women living alone

The self-transcendence enhancement program for the well-being of elderly women living alone was conducted across eight sessions (total of eight weeks). In the first session, participants were divided into small groups of 6 or 7 people, with each group given a name, and an introduction was provided to foster a comfortable and intimate group atmosphere. From the second session onward, the participants shared their impressions and encouragement about homework and weekly home activities, which were designed to introduce topics and activities from the next session to capture participants’ interest. First, body scan meditation was performed for 5 minutes, followed by specific activities for 35 minutes. The theme of the first session was “expressing and respecting oneself.” The second session’s theme was “focusing positively on life in the present free from negative feelings about regrets from the past.” The theme of the third session was “caring for oneself,” followed by “forgiving people in the past to focus on life in the present” in the fourth session. The theme of the fifth session was “maintaining intimate relationships with people with a heart full of gratitude for others,” and the theme of the sixth session was “living an unselfish life of helping others.” The seventh session’s theme was “having a good death” and the eighth session’s theme was “having purpose and meaning in life.” Activities were carried out individually or through interaction and participants’ experiences were shared. At the conclusion, the facilitator summarized the topics and activities of each session, and the participants shared their feelings about the activities. At this time, participants were given assignments to review concepts and themes related to the activities. For family activities, one theme was chosen among the following activities; reading, observing, religious activities, encountering nature, and meditation. The selected activity was to be practiced by participants to maintain self-transcendence activities during the week (Table 1).

### Instruments

The measurement tools used for data collection in this study were approved for use by the original authors.

#### Self-transcendence

The Korean version of the self-transcendence scale by Reed [25], of which the reliability and validity were verified by Kim et al. [26], was used. This tool inverted one item (item 15) and had a total of 15 items that were completed using a 4-point Likert scale (1 not at all, 4 strongly agree). The possible score range is 15-60 points, with a higher score indicating a higher degree of self-transcendence. Cronbach’s α was .80 at the original development of the tool [25], .85 in the Korean version developed by Kim et al. [26], and .93 in this study.

#### Spiritual well-being

The modified Korean version of the spiritual well-being scale [27] by Lee [28] was used in this study. This scale is composed of two subscales: religious spiritual well-being (10 items) which measures one’s relationship with God and satisfaction with human life; and existential spiritual well-being (10 items) which focuses on the meaning and purpose of life. The subscales are completed using a 6-point Likert scale (1, not at al; 6, strongly agree) with nine items being scored inversely. In this study, the 6-point Likert scale was converted to a 4-point Likert scale (1, not at al; 4, strongly agree) to make it easier for the participants to give their responses. The possible summed score range was 20-80 points, with a higher score indicating a higher degree of spiritual well-being. The original tool [27] had good internal consistency, i.e., Cronbach's α overall, .96 for the religious spiritual well-being subscale, and .86 for the existential spiritual well-being subscale. In Lee’s study [28], Cronbach’s α was .93 overall, .95 for the for the religious spiritual well-being subscale, and .86 for the existential spiritual well-being subscale. In this study, Cronbach’s α was .87 overall, .79 for the religious spiritual well-being subscale, .82 for the existential spiritual well-being subscale.

Psychological well-being is a subjective psychological state of health, which includes one’s ability to experience subjective satisfaction, euphoria, or general emotions and emotions when responding to environmental stimuli in daily life [29]. Therefore, in this study, depression, life satisfaction, and positive and negative emotions were measured as factors related to psychological well-being using the below tools.

#### Depression

Kee’s Korean version [30] of the geriatric depression scale (short version) developed by Sheikh and Yesavage [31] was used. This is a binomial response tool (0 no, 1 yes) and 6 out of 15 items (items 1, 5, 7, 11, 13, 15) are inverted. Out of a possible score of 0-15, the screening cutoff for elderly is 5 points [30]. For the original tool [31], Cronbach’s α was .94. It was .88 in the study of Kee [30] and .81 in this study.

#### Satisfaction with life

Kwak’s Korean version [32] of the satisfaction with life scale developed by Diener et al. [33] was adopted for this study. The five items are rated on a 5-point Likert scale (1, not at all; 5, strongly agree) with a possible score range of 5-25. A higher score indicates a higher level of life satisfaction. Cronbach’s α was .87 in the original tool [33], .80 in Kwak’s study [32], and .90 in this study.

#### Positive and negative affect

Jeon’s Korean version [34] of the positive and negative affect scale based on the original scale by Watson et al. [35] was used. It includes 20 items in total—nine on positive emotions and 11 on negative emotions—rated on a 5-point Likert scale (1 not at all, 5 strongly agree). Higher scores indicate higher levels of positive affect (possible score range, 9-45) or negative affect (possible score range, 11-55). The reliability of the original tool [35] was shown by Cronbach’s α of .86 for positive affect and .87 for negative affect. In the study by Jeon [34], it was .84 for positive affect and .79 for negative affect, and in this study, it was .96 for positive affect and .97 for negative affect.

### Procedures

This study was conducted from December 31, 2019 to February 23, 2020. The procedures and methods are described below.

#### Research assistant training and safety activities for research sites

The research assistants included two nursing students. They received education about the purpose of this study, the design and content of the program, research procedures and precautions, and the roles of research assistants. To keep the participants comfortable and safe, a welcoming indoor environment with sitting desks that could accommodate 20 people was prepared, risk factors related to falls (pointed objects, furniture with sharp corners) were rearranged, and regular safety checks were conducted. Protocols to protect study participants from coronavirus disease 2019 (COVID-19) were designed and followed. Researchers and assistants wore masks that covered their mouths and noses, remained at least 1 meter apart from others, washed their hands often with soap and water, and avoided crowds and poorly ventilated indoor spaces.

#### Pretest

A pretest survey was conducted after the researcher explained the purpose and method of the study to the 40 initial participants. The preliminary investigation period was from December 31, 2019 to January 2, 2020. The study participants gathered at the same time and filled out questionnaires for about 1 hour. The selection criteria required participants to be able to read, write, hear, comprehend, and express themselves in words, so there were no difficulties completing the questionnaires themselves; however, the participants had difficulties comprehending the response method. One researcher and two research assistants helped them with explanations.

#### Experimental intervention

The eight-session program was conducted across 8 weeks from January 3, to February 21, 2020, at two senior citizen centers with 40 participants. This period was prior to COVID-19 outbreaks in the area and infection protocols were not as rigid. Safety protocols were maintained as sessions were conducted, once per week for 1 hour each.

#### Posttest

A posttest survey was conducted from February 22, 23, 2020, after the completion of the 8-week program. The same measurement tools used in the pretest survey were used except for general characteristics, which were not collected in the posttest survey. This study followed the Transparent Reporting of Evaluations with Nonrandomized Designs (TREND) statement [36] to improve the reporting quality of nonrandomized evaluations of behavioral interventions.

#### Statistical analysis methods

The general characteristics of the participants were analyzed by frequency and percentage, mean, and standard deviation. To analyze the effect of the self-transcendence enhancement program, the paired t-test was conducted to analyze pre- and postintervention values. Statistical analysis was performed using IBM SPSS Statistics ver. 25.0 (IBM Corp., Armonk, NY, USA).

## Results

### General characteristics of the participants

The average age of the 34 participants was 79.10 years, 23 (67.6%) did not graduate from elementary school, and the average period for which they had lived alone was 10.08 years. The average monthly income of the participants was 497,400 Korean won (439 US dollars), and 25 people (73.5%) answered “poor” or “very poor” for their perceived health condition. Twenty-eight participants (82.3%) said their children were their guardians, while 4 (11.8%) listed relatives and 2 (5.9%) listed neighbors as their guardians. Fourteen participants (41.2%) were contacted by their guardians once per month, and 15 (44.1%) were visited by their guardians once per month. For leisure activities, 22 participants (64.7%) reported not having hobbies or participating in leisure activities, and 23 participants (67.6%) reported not being religious (Table 2).

### Evaluation of the applicability of the self-transcendence enhancement program

#### Effectiveness of the program

Participants’ self-transcendence, spiritual well-being, and psychological well-being increased after the intervention program (Table 3). The self-transcendence scores of elderly women living alone increased significantly from 43.26 points before participation in the program to 48.91 points after the program (t=8.78, *p*<.001). The participants’ overall spiritual well-being after the program increased significantly, from 56.52 points to 64.29 points (t=8.30, *p*=.002), and both the religious and existential spiritual well-being subareas also improved. The average religious spiritual well-being score increased from 27.76 points to 29.70 points after the program (t=1.79, *p*=.040), and the average existential spiritual well-being score increased from 29.55 points to 34.58 points (t=6.75, *p*=.002). Thus, the first and second hypotheses were accepted.

Positive affect increased, and depression and negative affect decreased. The participants’ average score for depression decreased from 8.73 points to 7.00 points after the program (t=–7.59, *p*<.001), and the average negative affect score decreased from 34.11 points before the program to 30.26 points after the program (t=–6.15, *p*<.001). In addition, the mean positive affect score rose from 30.41 points to 32.00 points (t=3.77, *p*=.001). The average life satisfaction score also increased from 14.14 points to 14.97 points, but it was not statistically significant (t=1.72, *p*=.470). Therefore, three out of the four components of the third hypothesis—decreased depression, increased positive affect, and decreased negative affect after the program—were accepted. However, the hypothesis that life satisfaction would increase was not accepted.

#### Adherence and acceptability

The attendance rate was 85%, with 34 people completing the program, and the attendance rate increased as the sessions proceeded after the first half, with a more than 90% attendance rate for the fourth session. Participants showed high satisfaction in the process of actively exchanging opinions during small-group activities, proactively exchanging their thoughts about their own experiences and feelings. During the program, there were no incidents such as damaged relationships or inappropriate disclosure of confidential information due to group activities across the 8 weeks. The main reasons that six people discontinued participation in the program were health problems (n=2), moving to another city (n=1), and not meeting the attendance criteria (n=1). In addition, there were no incidents related to the participants’ health or safety such as hypoglycemia, dehydration, or falls.

## Discussion

The improvement of the average self-transcendence score after the program is consistent with the results of another study [11] in which the self-transcendence score increased after a self-transcendence program for elderly women in the United States. This is likely due to the effect of the program on changing participants’ inner perspectives and worldviews as well as participants’ desires for a better life by achieving self-transcendence.

The average scores for the spiritual well-being of elderly women living alone were similar to those of underprivileged older women living alone [2] and higher than those of elderly people living alone in rural areas [37]. After participation in the program, participants experienced improvements in both existential and religious spiritual well-being. In particular, scores for existential spiritual well-being increased two times more than religious spiritual well-being. The reason for this is that other activities not related to religion were performed during the program, such as activities to help others, activities to face death, and activities to search for the purpose and meaning of the rest of their lives, to help participants live a harmonious life. Another study found that a self-transcendence program [4] reduced community-dwelling elderly people’s death anxiety and encouraged them to pursue lives led by meaning and purpose. Thus, these programs have been shown to improve participants’ sense of religious and existential spiritual well-being.

The average depression score reported by elderly women living alone in this study was higher than that found in another study of elderly women living alone [1,38], and more than twice that of community-dwelling elderly people [39]. The results of the present study support previous finding [4] that depression was influenced by economic status, education level, marriage status, health status, and social life. Participant’s depression levels were found to have decreased after participating in the program.

The average positive and negative affect scores after the program were 3.37 points and 3.10 points (out of 5 points), respectively. The average positive and negative affect scores were lower in this study than in a study of community-dwelling elderly people [40]. The program was effective at improving the affect of elderly women living alone. In particular, the average negative affect score decreased significantly. Therefore, ways to improve positive affect should be explored by devising a positive psychology program suitable for elderly women living alone, with a particular focus on low-income families [3].

However, the intervention program did not significantly affect participants’ life satisfaction. This is contrary to another study [11], which found that life satisfaction among home-dwelling elderly women who participated in a self-transcendence enhancement program significantly increased. The reason for this discrepancy may be that life satisfaction among the elderly is affected by monthly average income, education level, subjective health level, and other related factors [41], and there is a limit to how much life satisfaction could be improved through a single 2-month program.

There is a limitation to generalizing the present results to other regions and countries since we recruited elderly women living alone in a particular region. However, their physical, economic, and social demographic characteristics can be generalized. In addition, this study used a single-group pretest-posttest experimental design to evaluate the applicability of the program. The ability to evaluate the effectiveness of the program was also limited by the inability to control for confounding variables and maturation issues. Lastly, the researcher and two research assistants conducted and analyzed the program simultaneously. Therefore, the validity of the study is limited. In future studies, single-blind randomization should be used to generate more credible results by establishing both a control group and an experimental group.

The self-transcendence enhancement program for the well-being of elderly Korean women living alone developed in this study had a positive effect on spiritual (religious and existential) well-being and psychological well-being (depression, positive affect, and negative affect), and also enhanced participants’ sense of self-transcendence. Through this study, it was confirmed that this program could be safely conducted with other groups of elderly people. In addition, this study contributed to the body of evidence-based nursing knowledge related to promoting self-transcendence in elderly women living alone through the application of an intervention program based on nursing theory. The safety of this program was confirmed, and the attendance rate of the participants was satisfactory. Therefore, it is highly recommended that this program be conducted for small groups of people in the same age group at other institutions. Furthermore, nurses working in local communities should utilize connections between senior citizen centers and public health centers to implement this program for elderly women living alone.[Fig f3-kjwhn-2021-06-07]

## Figures and Tables

**Table 1.** Design of the self-transcendence enhancement program for elderly women living alone

Week	Theme [dimension]	Activities and contents	Duration (minute)	Homework
1	Respecting and expressing oneself	•Making group names and self-introduction	10	Praising each other’s appearance
		•Body scan meditation	40	
	[Intrapersonal]	•Expressing positive statements about one’s appearance		
		•Decorating one’s name tag to express oneself		
		•Activities summary, sharing thoughts, feedback	10	
2	Escaping from negative feelings of past regret and focusing positively on life in the present	•Sharing weekly homework and home activities	10	Writing about hobbies I want to do
				
	[Intrapersonal Temporal]	•Body scan meditation	40	
		•Drawing my life trajectory, talking about joys and sorrows		
		•Exploring negative feelings about past regrets		
		•Talking about the influence of negative feelings on daily life, the possibility of changing circumstances and actions in one’s past life, and something one wants to do to live a life without regrets		
		•Activities summary, sharing thoughts, feedback	10	
3	Taking care of one’s inner self	•Sharing weekly homework and home activities	10	Practicing inner-care activities
				
	[Intrapersonal Temporal]			
		•Body scan meditation	40	
		•Expressing gratitude to oneself using life trajectories		
		•Exploring ways to take care of one’s inner self		
		•Talking about the impact on one’s life and changes in perspectives on life when doing inner-care activities		
		•Activities summary, sharing thoughts, feedback	10	
4	Forgiving people from one’s past	•Sharing weekly homework and home activities	10	Writing a letter of forgiveness
				
	[Transpersonal Temporal]	•Body scan meditation	40	
		•Expressing one’s negative feelings about hurtful experiences using life curves and talking about the influence of those feelings on daily life		
		•Facilitator’s education: importance and methods of forgiveness		
		•Writing a forgiveness resolution to a person to forgive him or her		
		•Activities summary, sharing thoughts, feedback	10	
5	Maintaining close relationships with people	•Sharing weekly homework and home activities	10	Delivering a gift box to someone
				
	[Intrapersonal Interpersonal]	•Body scan meditation	40	
		•Drawing and introducing a map of one’s social network		
		•Talking about positive experiences within social networks and how to restore estranged relationships		
		•Making a gift box for someone I would like to be close to again		
		•Activities summary, sharing thoughts, feedback	10	
6	Living an altruistic life by helping others	•Sharing weekly homework and home activities	10	Practicing group volunteer activities
				
	[Interpersonal Transpersonal]	•Body scan meditation	40	
		•Watching videos of senior citizens doing volunteer work		
		•Facilitator’s education: the meaning and value of volunteering and positive benefits to life		
		•Sharing volunteer work experiences (feelings, motivation)		
		•Presentation after discussion of practical group volunteer activities		
		•Activities summary, sharing thoughts, feedback	10	
7	Greeting a good death	•Sharing weekly homework and home activities	10	Writing my daily life list in preparation for a good death
				
	[Transpersonal Temporal]	•Body scan meditation	40	
		•Watching videos: older people living meaningfully to greet a good death		
		•Facilitator’s education: recognizing death as a part of life, preparation for a good death, living meaningfully for the rest of life		
		•Sharing one’s death anxiety, what to do to prepare for a good death		
		•Making a will or writing a will for family		
		•Activities summary, sharing thoughts, feedback	10	
8	Seeking purpose and meaning in life	•Sharing weekly homework and home activities	10	Practicing
		•Introducing session theme and activities		Lessons from the 8-week program in daily life
	[Transpersonal Temporal]	•Drawing my own tree of hope: future plans and goals, life purpose and meaning	40	
		•Presentation of life plans, goals, and meaning using the hope tree		
		•Sharing thoughts on completing the 8-week program	10	

**Table 2.** General characteristics of participants (N=34)

Characteristics	Categories	n (%)	Mean ± SD
Age (year)	75–80	19 (55.9)	79.10 ± 2.8
	81–84	15 (44.1)	
Education level	<Elementary school	23 (67.6)	
	≥Elementary school	11 (32.4)	
Time spent living alone (year)	≤5	4 (11.8)	10.08 ± 3.74
	6–10	19 (55.8)	
	11–15	7 (20.6)	
	≥16	4 (11.8)	
Monthly income (KRW)	≤3 million	10 (29.4)	49.74 ± 15.39
	3.1-5.9 million	16 (47.1)	
	≥6 million	8 (23.5)	
Perceived economic status	Adequate	5 (14.7)	
	Insufficient	15 (44.1)	
	Very bad	14 (41.2)	
Perceived health status	Good	9 (26.6)	
	Poor	23 (67.6)	
	Very poor	2 (5.9)	
Guardian type	Children	28 (82.3)	
	Relatives	4 (11.8)	
	Neighbors	2 (5.9)	
Frequency of contact with guardian	Once a week	13 (38.2)	
	Once a month	14 (41.2)	
	More than once a month	7 (20.6)	
Frequency of visits by guardian	Once a week	6 (17.7)	
	Once a month	15 (44.1)	
	More than once a month	13 (38.2)	
Hobbies	Singing classes	3 (8.8)	
	Religious activities	5 (14.7)	
	Exercise and walking	4 (11.8)	
	None	22 (64.7)	
Religion	Yes	11 (32.4)	
	No	23 (67.6)	

KRW: Korean won (1 million KRW is approximately 900 US dollars)

**Table 3.** The effect of the self-transcendence enhancement program on study outcomes (N=34)

Variable	Possible score range	Mean ± SD		t	*p* (one-tailed)
		Pretest	Posttest		
Self-transcendence	15-60	43.26 ± 7.47	48.91 ± 7.82	8.78	<.001
Spiritual well-being	20-80	56.52 ± 8.09	64.29 ± 7.82	8.30	.002
Religious spiritual well-being	10-40	27.76 ± 7.76	29.70 ± 7.52	1.79	.040
Existential spiritual well-being	10-40	29.55 ± 5.95	34.58 ± 4.95	6.75	.002
Psychological well-being					
Depression	0-15	8.73 ± 1.83	7.00 ± 1.49	–7.59	<.001
Life satisfaction	5-25	14.14 ± 4.66	14.97 ± 4.87	1.72	.470
Positive affect	9-45	30.41 ± 7.42	32.00 ± 6.91	3.77	.001
Negative affect	11-55	34.11 ± 4.86	30.26 ± 4.47	–6.15	<.001

## Supplementary materials

Further details on supplementary materials are presented online (available at https://doi.org/10.4069/kjwhn.2021.06.07).
