# Supplementary material for: Development and application of a self-transcendence enhancement program for the well-being of elderly women living alone in Korea
Source: Korean J Women Health Nurs. 2021 Jun 24;27(2):128–40. doi: 10.4069/kjwhn.2021.06.07 (PMC9334186; doi:10.4069/kjwhn.2021.06.07)
Supplement: Supplementary file 1 [file kjwhn-2021-06-07-suppl.pdf]

Supplementary data. In-depth interview questions

1st interview

1) 귀하의 삶이 어떠했는지 과거부터 살아온 과정과 모습을 말해 주세요?
   Tell me what life was like and how you've lived in the past.

2) 귀하는 요즈음 무엇으로 소일거리를 하고 계십니까?
   What do you do to pass time these days?

3) 귀하는 주변 사람들과 어떻게 지내고 계십니까?
   How are you getting along with the people around you?

4) 귀하는 요즈음 걱정되거나 불편한 점이 있으면 말씀해 주세요?
   Can you tell me if you have any concerns or uncomfortable issues these days?

2nd interview

1) 귀하에게 가족은 어떤 의미를 가지고 있습니까?
   What does family mean to you?

2) 귀하는 요즈음 삶의 과정에서 드는 감정들이 어떠하십니까?
   How do you feel about the course of life these days?

3) 귀하는 지금의 모습대로 나이 들어가는 것에 대해 어떤 생각이 드십니까?
   How do you feel about getting old the way you are now?

4) 귀하는 초월적인 존재(절대자, 신)과의 관계에서 삶을 위한 힘과 지지를 얻고 있습니까? 또는 귀하의 삶의 목적과 의미를 이끄는 힘은 무엇입니까?
   Do you have the strength and support for life in your relationship with transcendent beings? Or what is the power that drives the purpose and meaning of your life?

5) 귀하가 좀 더 나은 삶을 위해 본인의 상황에서 어떤 것이 필요하다고 생각하십니까?
   What do you think you need in your situation for a better life?

https://doi.org/10.4069/kjwhn.2021.06.07
